# Perturbation with Intrabodies Reveals That Calpain Cleavage Is Required for Degradation of Huntingtin Exon 1

**DOI:** 10.1371/journal.pone.0016676

**Published:** 2011-01-31

**Authors:** Amber L. Southwell, Charles W. Bugg, Linda S. Kaltenbach, Denise Dunn, Stefanie Butland, Andreas Weiss, Paolo Paganetti, Donald C. Lo, Paul H. Patterson

**Affiliations:** 1 Division of Biology, California Institute of Technology, Pasadena, California, United States of America; 2 Center for Drug Discovery, Department of Neurobiology, Duke University Medical Center, Durham, North Carolina, United States of America; 3 Centre for Molecular Medicine and Therapeutics, Child and Family Research Institute, University of British Columbia, Vancouver, British Columbia, Canada; 4 Neuroscience Discovery, Novartis Institutes for BioMedical Research, Novartis Pharma AG, Basel, Switzerland; Brigham and Women's Hospital, Harvard Medical School, United States of America

## Abstract

**Background:**

Proteolytic processing of mutant huntingtin (mHtt), the protein that causes Huntington's disease (HD), is critical for mHtt toxicity and disease progression. mHtt contains several caspase and calpain cleavage sites that generate N-terminal fragments that are more toxic than full-length mHtt. Further processing is then required for the degradation of these fragments, which in turn, reduces toxicity. This unknown, secondary degradative process represents a promising therapeutic target for HD.

**Methodology/Principal Findings:**

We have used intrabodies, intracellularly expressed antibody fragments, to gain insight into the mechanism of mutant huntingtin exon 1 (mHDx-1) clearance. Happ1, an intrabody recognizing the proline-rich region of mHDx-1, reduces the level of soluble mHDx-1 by increasing clearance. While proteasome and macroautophagy inhibitors reduce turnover of mHDx-1, Happ1 is still able to reduce mHDx-1 under these conditions, indicating Happ1-accelerated mHDx-1 clearance does not rely on these processes. In contrast, a calpain inhibitor or an inhibitor of lysosomal pH block Happ1-mediated acceleration of mHDx-1 clearance. These results suggest that mHDx-1 is cleaved by calpain, likely followed by lysosomal degradation and this process regulates the turnover rate of mHDx-1. Sequence analysis identifies amino acid (AA) 15 as a potential calpain cleavage site. Calpain cleavage of recombinant mHDx-1 *in vitro* yields fragments of sizes corresponding to this prediction. Moreover, when the site is blocked by binding of another intrabody, V_L_12.3, turnover of soluble mHDx-1 in living cells is blocked.

**Conclusions/Significance:**

These results indicate that calpain-mediated removal of the 15 N-terminal AAs is required for the degradation of mHDx-1, a finding that may have therapeutic implications.

## Introduction

Huntington's disease (HD) is caused by the expansion of a polyglutamine (polyQ) tract in the first exon (HDx-1) of the large protein, huntingtin (Htt) [1]. Mutant Htt protein (mHtt) perturbs many cellular processes by both gain of toxic function and loss of normal function. These include axonal transport, mitochondrial metabolism, transcriptional regulation and the ubiquitin proteasome system (UPS) [2]. There is an age-dependent accumulation of mHtt protein in HD [3], which may be partially responsible for the adult onset of symptoms despite the lifelong expression of mHtt. Increasing the clearance of mHtt could prevent this accumulation and thereby delay or prevent the onset of symptoms.

Degradation of mHtt occurs through several mechanisms, suggesting a number of potential therapeutic opportunities for enhancing removal. Proteases cleave Htt, generating N-terminal fragments, some of which are more toxic than the full-length protein [4], [5], [6]. Increasing polyQ tract length leads to increased caspase and calpain activation and enhanced production of toxic N-terminal fragments in the HD brain [7]. These fragments are degraded by additional protease cleavage, the UPS and autophagy, which can involve isolation in an autophagosome and introduction to the lysosome by fusion, macroautophagy, or delivery to the lysosome by chaperone proteins (chaperone-mediated autophagy, CMA) [8]. Certain cleavage events generate toxic fragments, and selective prevention of these events dramatically reduces the toxicity of mHtt by the generation of other, less toxic N-terminal cleavage products [9], [10]. Post-translational modifications such as phosphorylation also play a role in regulating Htt proteolysis [11], [12], and phosphorylated mHtt can be more toxic than unphosphorylated mHtt [12]. Thus, the dichotomy of mHtt processing: while some modifications increase the toxicity of the protein, these more toxic forms are intermediates in the process leading to total degradation. Since enhancing total degradation represents a powerful therapeutic strategy, a better understanding of this process is warranted. As the site of the disease-causing mutation, insight into the clearance of HDx-1 is particularly salient.

We have used intrabodies (iAbs), intracellularly-expressed antibody fragments directed against various sites in HDx-1 to gain such insight. Intrabodies retain the high target specificity of antibodies but lack the immunogenic constant domains. These reagents have shown significant promise as therapeutics for proteinopathies including HD [13]. Moreover, iAbs are also powerful molecular tools for probing the functions and interactions of their targets when expressed in living cells.

We have previously shown that binding of the iAb Happ1, which recognizes the proline rich region of HDx-1, results in a selective increase in the turnover of the mutant form (mHDx-1) [14], [15]. Here we report on the mechanism of Happ1-induced turnover of mHDx-1, the study of which has revealed a new insight into mHtt cleavage.

## Materials and Methods

### Cell culture

HEK 293 cells (ATCC) and ST14A cells (Elena Cattaneo, Milano, Italy) were grown in DMEM (Invitrogen) supplemented with 10% heat inactivated fetal bovine serum, 2 mM glutamine, 1 mM streptomycin and 100 international units of penicillin (Invitrogen). Cells were maintained in 37°C (293) or 33°C (ST14A) incubators with 5% CO_2_. Transfections utilized calcium phosphate.

### Ubiquitination of Htt

HEK 293 cells were transfected with mHDx-1-GFP plus iAb (HDx-1:iAb  =  V_L_12.3, 1∶1; Happ1, 1∶2). Thirty-six hours post-transfection, cells were collected for Western blotting and immunoprecipitation (IP) as previously described [14]. Briefly, cells were dislodged by pipetting, pelleted by centrifugation, rinsed with PBS, and lysed by sonication in lysis buffer. Insoluble material was removed by additional centrifugation, and the protein concentration was determined by BCA assay (Pierce). Htt protein was immunoprecipitated from the lysate by combining 400 µg lysate protein with 50 µg anti-GFP antibody (Invitrogen) conjugated to protein G sepharose beads (Sigma) and rocking for 4 hrs at RT. Beads were washed 4 times in PBS containing 0.1% Triton X100 to remove unbound protein. Seventy-five µg total lysate protein samples and bound IP samples were boiled in 6X protein loading buffer containing 20% β-mercaptoethanol (BME), separated by polyacrylamide gel electrophoresis (PAGE), transferred to nitrocellulose membrane, and immunoblotted for ubiquitin. Membranes were then stripped with Restore Western blot stripping buffer (Pierce) and re-blotted for Htt. Membranes were stripped a second time and immunoblotted for β-tubulin, used as a loading control. The ratio of immunoprecipitated ubiquitin (ubiquitinated Htt) to immunoprecipitated Htt (total Htt) was assessed using chemiluminescence densitometry. Each band was first normalized to the density of the β-tubulin band for that sample. Immunoprecipitated Htt and ubiquitin levels were compared by computing the ratio of the density of the band in the presence of Happ1 to the density of the band in the presence of V_L_12.3. The ratio of immunoprecipitated ubiquitin:Htt in the presence of Happ1 vs. V_L_12.3 was then compared. The experiment was repeated two additional times, giving an N of 3.

### Htt levels

HEK 293 cells were transfected with mHDx-1-GFP plus iAb (V_L_12.3, 1∶1; Happ1, 2∶1) or mHDx-1ΔPRR (lacking the proline-rich region) plus iAb as a control for non-specific effects of Happ1. Inhibitors of proteolytic processing were added to the culture medium 24 hrs post-transfection in the following concentrations: lactacystin (Sigma), 10 µM; epoxomicin (Sigma), 10 µM; 3-MA (Sigma), 10 mM; bafilomycin A1 (Sigma), 100 nM; caspase inhibitor I (NBD Biosciences), 50 µM; calpain inhibitor I (Sigma), 50 µM. Cell lysates were prepared for Western blotting 48 hrs post-transfection as described above. Membranes were blotted for Htt, and stripped and blotted for β-tubulin as a loading control. Htt levels were compared by chemiluminescence densitometry. The density of each band was normalized to the density of the β-tubulin band for that sample. The ratio of Htt levels in the presence of Happ1 to Htt levels in the presence of V_L_12.3 was then compared. To reduce the likelihood that observed differences in Htt level resulted from differences in transfection efficiency, the experiment was repeated 3 times, giving an N of 4.

### Htt turnover

Htt turnover was assayed using the SNAP-tag fusion system for *in vivo* protein labeling (NEB) as previously described [14]. Briefly, ST14A cells were grown on cover slips and transfected with mHDx-1-SNAP alone or with iAb. To control for non-specific effects of Happ1, mHDx-1ΔPRR–SNAP alone or with iAb was used. Green fluorescent SNAP-substrate was added to cells 24 hrs post-transfection to label all mHDx-1. Immediately after labeling, some samples were fixed in 4% paraformaldehyde (PFA), stained for cell nuclei (topro-3-iodide, Invitrogen) and mounted for confocal microscopy. Inhibitors of proteolytic processing were then added to the medium of the remaining cultures. At 48 hrs post-transfection, these cells were fixed, stained and mounted as above. Mean intensity of green fluorescence in cells in three microscope fields per well and three wells for each condition was used to determine average Htt levels. The ratio of mean cell intensity at 24 hrs to mean cell intensity at 48 hrs was computed to determine rate of Htt turnover. The experiment was repeated twice, giving an N of 3.

### Calpain cleavage site prediction in HDx-1

Calpain 1 and calpain 2 cleavage sites in HDx-1 were predicted using the SitePrediction tool with the pre-computed cleavage site profiles “calpain-1_Homo_sapiens_4_2” and “calpain- 2_Homo_sapiens_4_2” [16]. The HDx-1 sequence was obtained from the February 2009 human reference sequence (GRCh37) through the UCSC Genome Browser [17]. PolyQ tract lengths of 17 and 42 AAs were used to determine if cleavage is modified by polyQ length.

### *In vitro* calpain cleavage of HDx-1

HDx-1 Q46 fused to a thioredoxin tag (TRX) to promote solubility (mHDx-1-TRX) was purified as reported previously [18] and exchanged into diluent (20 mM Tris-HCl, pH 7.4 containing 150 mM NaCl and 2 mM Ca2^+^) using a disposable PD10 desalting column (GE Healthcare). 20 µg purified mHDx-1-TRX was incubated with 1 µg calpain 1 (Sigma) at 37°C for 1 hour. As a control for possible calpain cleavage in the TRX tag or linker sequence, mHDx-1-TRX was cleaved with enterokinaseMax (EKMax) (Invitrogen), a peptidase known to remove the entire TRX-linker sequence from mHDx-1-TRX according to the manufacturer's specifications. Cleavage reactions were separated by PAGE and stained with coomassie to visualize protein bands. Molecular weight of protein bands was determined by comparison to precision plus dual protein molecular weight standard (Biorad) using a FluorChem 8900 (Alpha Innotech) gel documentation system and AlphaImager software. A reaction containing only mHDx-1-TRX and diluent was used to assess uncleaved protein and reactions containing either calpain 1 or EKMax and diluent in the absence of mHDx-1-TRX were used to visualize bands corresponding to these proteins. To identify cleavage fragments, a reaction containing mHDx-1-TRX alone and one containing mHDx-1-TRX and calpain were transferred to nitrocellulose membrane after PAGE separation and blotted with an antibody recognizing the N-terminus of Htt [19].

### Peptide array mapping of the V_L_12.3 binding site

A 14-mer 7 peptide array covering Htt N1-32 in steps of three AAs was purchased from Mimotopes. Peptides were dissolved in 80% DMSO/20% water to an average concentration of 5 mg/ml. Peptides were further diluted 100-fold in water and 2 µl of each peptide was blotted onto nitrocellulose and allowed to dry. Nitrocellulose membranes were blocked with 1% milk in PBS for one hour at room temperature. GST-V_L_12.3 generated as previously described [14] was added as primary antibody and incubated overnight at 4°C. After washing in PBS with 0.1% Tween, anti-HA tag antibody 3F10 coupled to HRP (Roche) was added for 1 hour at room temperature. Dot blots were developed by exposure to X-ray film.

### Organotypic brain slice cultures

Brain tissue was removed from euthanized postnatal day 10 (P10) CD Sprague-Dawley rats (Charles River Laboratory, Raleigh, NC) in accordance with Duke University Medical Center Institutional Animal Care and Use Committee guidelines and approvals (approval #A248-08-09), and as described previously [14], [20]. 250 µm thick coronal slices containing both striatum and cortex were cut using Vibratomes (Vibratome, St. Louis, MO) in ice-cold culture medium containing 15% heat-inactivated horse serum, 10 mM KCl, 10 mM HEPES, 100 U/ml penicillin/streptomycin, 1 mM MEM sodium pyruvate, and 1 mM L-glutamine in Neurobasal A (Invitrogen). Corticostriatal brain slices were then incubated at 37°C under 5% CO_2_ for 1 hr before biolistic transfection with 1.6 µm gold particles coated with DNA constructs expressing yellow fluorescent protein (YFP) as a vital marker, mHDx-1 Q-73, and either V_L_12.3 or the CV_L_ non-targeting intrabody. For control transfections, gold particles were coated with YFP alone plus vector backbone DNAs. For time resolved Förster resonance energy transfer (TR-FRET) analysis, brain slices were triturated 10x through 26-gauge needles in ice-cold lysis buffer (50 mM Tris-HCl, 150 nM NaCl, 2 mM EDTA, 1% NP-40, 0.1% SDS + Roche protease inhibitor cocktail tablet) and centrifuged at 12,000 g for 10 min. at 4°C. The supernatants were collected and stored at −80°C until TR-FRET analysis. Experimental conditions were run in triplicate using a single brain slice per lysate.

### Primary neuron co-culture

Cortico-striatal co-cultures were prepared as described in [21]. Briefly, cortices and striata from embryonic day 18 (E18) rat brains were dissected then separately dissociated with papain/DNase (Worthington biochemicals). Dissociated striatal and cortical neurons were counted and 5×10^6^ cells each were separately co-transfected by nucleofection (Amaxa, Lonza AG) with plasmids encoding HDx-1 carrying 73 CAG repeats or empty vector and either V_L_12.3 or the preimmune control CV_L_. After electroporation, striatal and cortical cells were combined and 60,000 cells/well were plated onto an established bed of astroglia in 96-well plates. Astroglial feeder layers were generated by dissection of E18 cortices followed by three serial passages to establish an enriched population of astroglia. Astroglia were plated into 96-well plates at a density of 2000 cells/well three days before neuron plating. Neurons were cultured in Neurobasal media (Invitrogen, Carlsbad, CA) supplemented with 5% fetal calf serum (Sigma-Aldrich, St. Louis, MO), 2 mM glutamine (Glutamax, Invitrogen), 10 mM potassium chloride, and 5 µg/mL gentamicin at 37°C in 95% 0_2_/5%CO_2_ for 4–6 days before analysis.

For TR-FRET analysis, cells were harvested by scraping from wells and triturating as described above for brain slice explants.

### Soluble mHtt-TR-FRET assay

TR-FRET detection of soluble mHtt was previously described [22]. In brief, samples were lysed in PBS, 0.4% TritonX100 and Complete Protease Inhibitor (Roche). 5 µl sample plus 1 µl antibody mix diluted in assay buffer (50 mM NaH_2_PO_4_, 400 mM NaF, 0.1% BSA and 0.05% Tween) were pipetted per well of a low-volume 384 microtiter plate (Sigma). Final antibody amount per well was 1 ng 2B7-terbium cryptate-labeled antibody and 10 ng MW1-D2-labeled antibody. Plates were incubated for 1 h at 4°C and measured with a Xenon-lamp Envision Reader (PerkinElmer) after excitation at 320 nm. Signal measured at 620 nm resulted from the emission of the terbium cryptate-labeled antibody and was used as a normalization signal for possible assay artifacts due to scattering, quenching, absorption or sample turbidity. Mutant Htt specific signal which resulted from the time-delayed excitation of the D2-labeled MW1 antibody after excitation by the terbium cryptate was detected at 665 nm. 665/620 nm signal ratio was calculated as “TR-FRET signal” specific for soluble mutant Htt.

## Results

### Happ1 does not increase mHDx-1 ubiquitination

**Figure 1:**
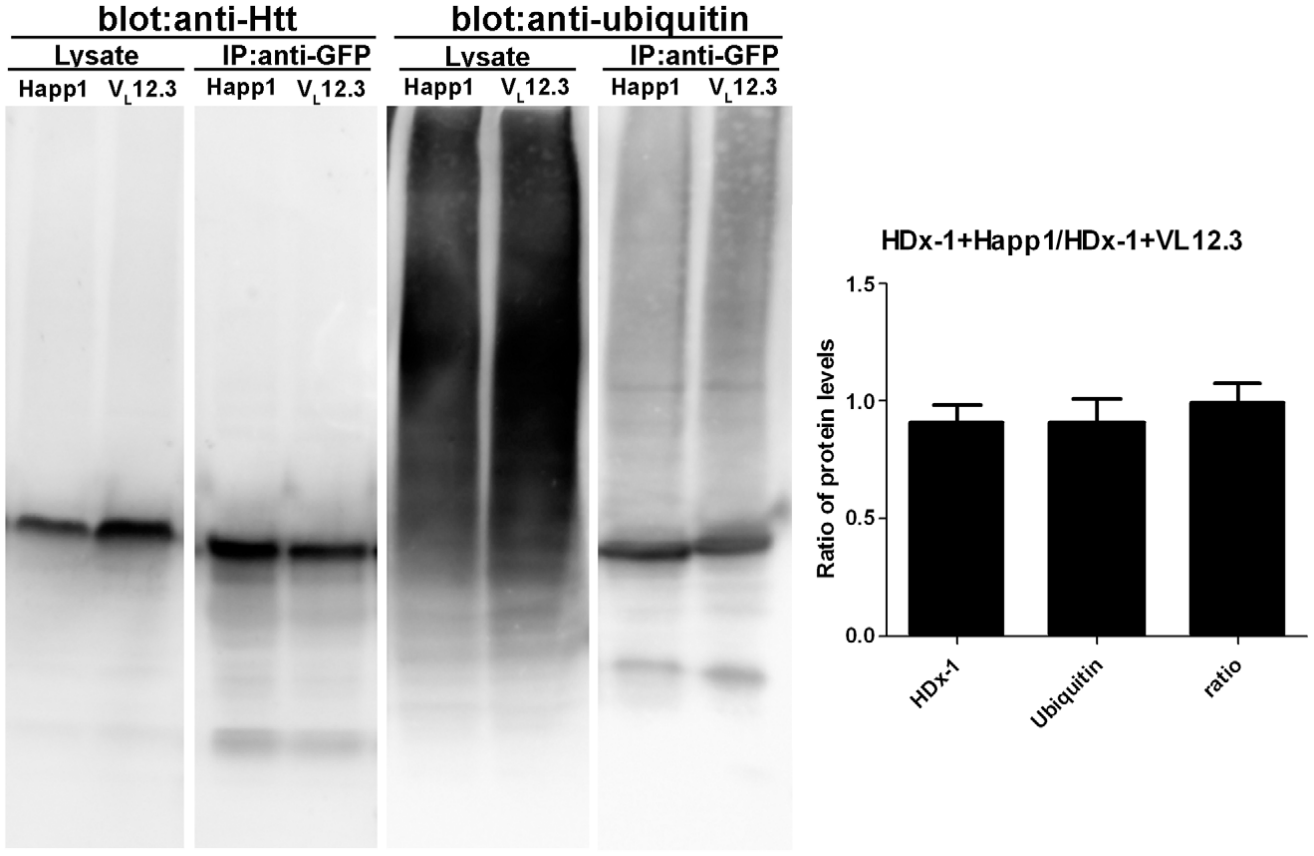
Happ1 does not increase ubiquitination of mHDx-1. mHDx-1 was immunoprecipitated from the lysates of HEK 293 cells co-transfected with mHDx-1 and iAb. (A) Lysates and IPs were Western blotted for Htt and ubiquitin. (B) The ratio of immunoprecipitated Htt (total mHDx-1) to immunoprecipitated ubiquitin (ubiquitinated mHDx-1) was compared. There are no iAb specific effects on this ratio. N = 3.

To assess the effects of Happ1 on ubiquitination of mHDx-1, HEK 293 cells were co-transfected with mHDx-1-GFP plus Happ1 or V_L_12.3. V_L_12.3 was used as a control for non-specific iAb effects as we have previously shown that this iAb binds mHDx-1 but has no effect on its levels in this system [14]. Huntingtin was immunoprecipitated from transfected cell lysates and immunoblotted for both Htt and ubiquitin (Fig. 1A). Densitometry was used to determine the ratio of ubiquitinated mHDx-1 to total mHDx-1 in the presence of Happ1 versus V_L_12.3 (Fig. 1B). There is no differential effect of iAb treatment on this ratio, indicating that Happ1 does not increase mHDx-1 ubiquitination and therefore likely does not work through a UPS-dependent mechanism.

### Happ1-induced reduction of mHtt levels requires calpain activity and maintenance of lysosomal pH

**Figure 2:**
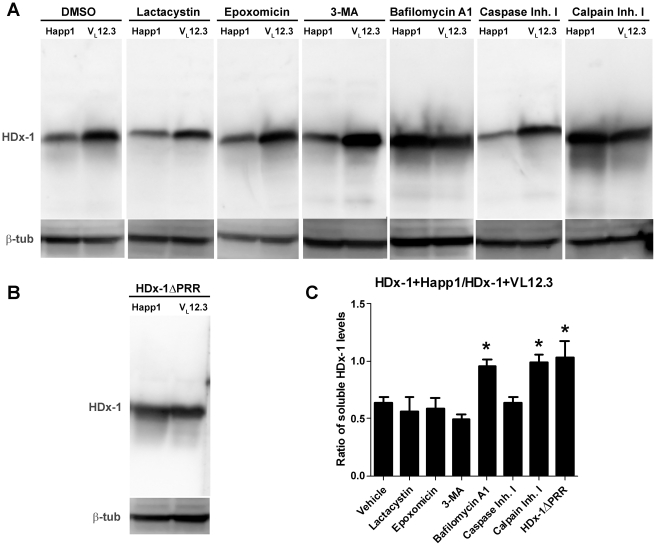
Happ1-mediated reduction of mHDx-1 protein levels is calpain-dependent. HEK 293 cells were co-transfected with mHDx-1 and iAb in the presence of inhibitors of proteolysis or DMSO vehicle. mHDx-1 protein levels in transfected cell lysates was compared by (A, B) Western blotting and (C) densitometry. There is less mHDx-1 protein in the Happ1 transfected cells as compared to the V_L_12.3 transfected cells in the presence of vehicle, Lactacystin, epoxomicin, 3-MA or caspase inhibitor 1. Happ1-mediated reduction of mHDx-1 levels is blocked by bafilomycin A1 or calpain inhibitor 1 to the same level as mHDx-1 lacking the Happ1 binding site. * = p<.05, N = 4.

We previously showed that Happ1 stimulates mHDx-1 turnover [14]. To determine which proteolytic pathway is involved, soluble lysates of HEK 293 cells co-transfected with mHDx-1 plus iAb, or mHDx-1ΔPRR plus iAb, and treated with various inhibitors of proteolytic processing were immunoblotted for Htt (Fig. 2A). The ratio of the Htt level in the presence of Happ1 to the Htt level in the presence of V_L_12.3 was compared among the various inhibitors. In unperturbed cells, or in the presence of DMSO vehicle, the level of Htt in the presence of Happ1 is reduced compared to the level of Htt in the presence of V_L_12.3. This ratio is unchanged by the addition of various inhibitors: lactacystin, a proteasome inhibitor that also affects cathepsin A of lysosomes; epoxomicin, a proteasome inhibitor; 3-MA, an inhibitor of autophagosome formation; or caspase inhibitor I, an irreversible pan-caspase inhibitor. In contrast, the addition of bafilomycin A1, an inhibitor of the vacuolar-type H(+)-ATPase that is known to inhibit autophagosome/lysosome fusion as well as lysosomal pH; or calpain inhibitor I, a pan-calpain inhibitor, significantly increases the ratio of Htt in the presence of Happ1 to Htt in the presence of V_L_12.3 (Fig. 2C). Thus, these latter two inhibitors interfere with the mechanism by which Happ1 reduces the level of mHtt. The levels of HDx-1 in the presence of calpain inhibitor I or bafilomycin A1 are quantitatively very similar to those found using the HDx-1 construct lacking the proline-rich region to which Happ1 binds (Fig. 2B). Thus, it appears that these inhibitors completely abolish the effect of Happ1 on mHDx-1 clearance. The effect of bafilomycin A1 is likely due to disrupted lysosomal pH rather than inhibition of autophagosome/lysosome fusion as evidenced by the lack of effect by 3-MA, which should act upstream of bafilomycin A1 in the macro-autophagy pathway. Therefore, we infer that Happ1 reduces mHDx-1 level by a calpain-CMA-dependent mechanism.

### Happ1-induced stimulation of mHtt turnover requires calpain activity and maintenance of lysosomal pH

**Figure 3:**
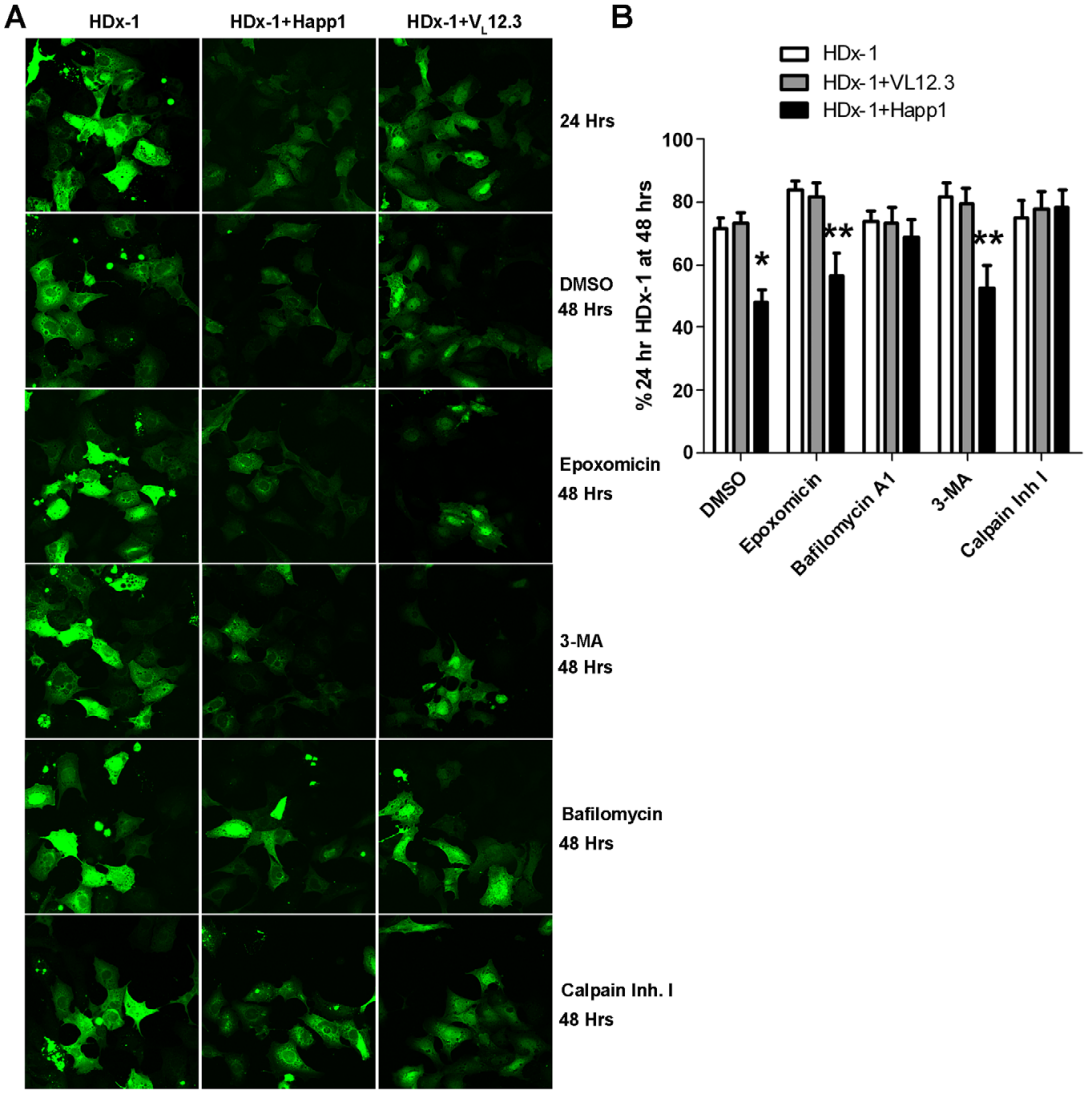
Happ1-enhanced mHDx-1 turnover is calpain-dependent. ST14A cells were co-transfected with mHDx-1-SNAP alone or with iAb in the presence of inhibitors of proteolytic processing or DMSO vehicle. To measure Htt turnover, mHDx-1 protein was labeled 24 Hrs post transfection and cultures were incubated for an additional 24 Hrs. (A) Immunofluorescent images showing labeled mHDx-1 (B) The mean cell intensity of label at 24 Hrs vs. 48 Hrs was used to determine the percentage of mHDx-1 labeled at 24 Hrs that still remained at 48 Hrs. In the presence of epoxomicin or 3M-A there is no change in Happ1-enhanced mHDx-1 turnover as compared to in the presence of DMSO. In the presence of bafilomycin A1 or calpain inhibitor 1, mHDx-1 turnover is not increased by Happ1. * = p<.05, ** = p<.01, N = 3.

In another approach to defining how Happ1 stimulates mHDx-1 clearance, mHDx-1 was labeled with the SNAP reagent and the loss of the label followed over time [23]. A traditional pulse chase experiment was not used because mHDx-1 is known to affect transcriptional regulation. This property of mHDx-1 could conceivably be altered by iAb binding, leading to variable transcription rates of HDx-1 in the presence of the various iAbs. The SNAP-tag fusion system allows labeling of all preexisting HDx-1. By measuring the amount of Htt at the time of labeling and again at a later time point, we are able to measure a rate of turnover independent of transcription or translation rate. This system also offers greater specificity, because only the SNAP-tag fusion protein is labeled as opposed to all cellular proteins translated during the labeling period as with traditional pulse-chase experiments. ST14A cells were transfected with mHDx-1-SNAP alone or with iAb, as well as mHDx-1ΔPRR-SNAP alone or with iAb. Green fluorescent SNAP substrate was used to label mHDx-1 protein 24 hrs post-transfection (Fig. 3A). Cells were allowed to incubate an additional 24 hrs in the presence of various inhibitors of proteolytic processing or vehicle. The mean fluorescence intensity of cells at 24 hrs and at 48 hrs was compared to determine the amount of mHDx-1 labeled at 24 hrs that still remained at 48 hrs (Fig. 3B).

Compared to that in the presence of V_L_12.3, there is significantly less mHDx-1 remaining at 48hrs in the presence of Happ1. Addition of epoxomicin or 3-MA has no effect on the turnover rate of mHDx-1 in the presence of Happ1, reinforcing the conclusion that Happ1 does not increase mHDx-1 turnover by enhancing proteasome or macroautophagy function. On the other hand, addition of bafilomycin A1 or calpain inhibitor I completely blocks the Happ1 stimulation of mHDx-1 turnover, leading to turnover levels equivalent to those with mHDx-1 alone or in the presence of V_L_12.3 (Fig. 3). These results support the finding with total HDx-1 levels (above) that Happ1 increases turnover of mHtt by enhancing calpain cleavage and CMA.

**Figure 4:**
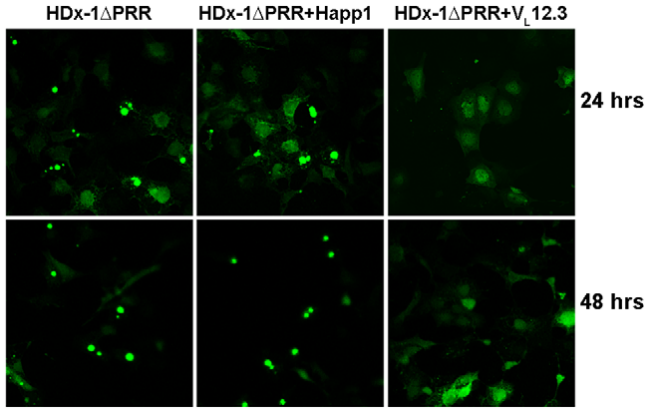
Happ1 does not inhibit aggregation of mHDx-1ΔPRR. ST14A cells were co-transfected with mHDx-1ΔPRR-SNAP alone or with iAb. HDx-1-SNAP fusion protein was labeled 24 Hrs post transfection, and labeled protein was observed 24 Hrs later. As expected, Happ1 has no effect on aggregation of mHDx-1 lacking the Happ1 binding site. Conversely, V_L_12.3 is still efficient at preventing aggregation of this modified mHDx-1.

A turnover rate could not be assessed for mHDx-1ΔPRR due to the increased toxicity and aggregation of this construct, leading to a paucity of morphologically normal cells or soluble HDx-1 at 48 hrs (Fig. 4). There is still significant soluble HDx-1 in the presence of V_L_12.3 at this time point, indicating that, as expected from our previous work, this iAb inhibits aggregation of this modified HDx-1, while Happ1 does not.

### There are putative calpain cleavage sites at AA 15 and AA 8 of HDx-1

To identify the site of calpain action, human HDx-1 amino acid sequence was analyzed for potential calpain 1 and 2 cleavage sites using the web application SitePrediction [16]. Using this program, AAs 12–17 with cleavage between 15 and 16, (ESLK.SF), is predicted to be the most likely site for both proteases, with greater than 99.9% specificity for calpain 1 and greater than 99% specificity for calpain 2. A secondary site at AAs 5–10, with cleavage between 8 and 9, (EKLM.KA), is predicted to have greater than 99% specificity for calpain 1 and greater than 95% specificity for calpain 2 (Fig. S1).

### Calpain 1 cleaves mHDx-1 *in vitro*

**Figure 5:**
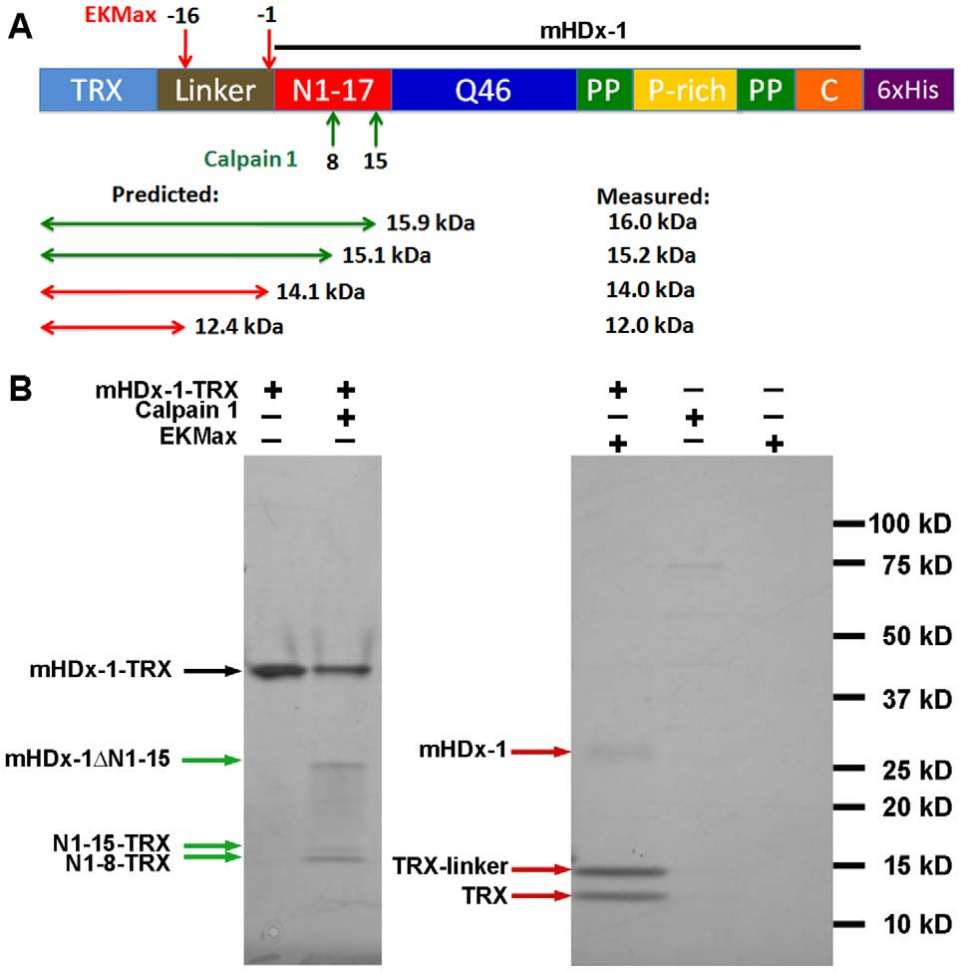
Purified calpain 1 cleaves HDx-1 *in vitro* generating cleavage fragments consistent with the predicted sites at AA8 and AA15. HDx-1 Q46 fused to thioredoxin (mHDx-1-TRX) was incubated with purified calpain 1 *in vitro*, separated by PAGE and stained with coomassie to assess cleavage. (A) mHDx-1-TRX construct showing known EKMax cleavage sites and predicted calpain 1 cleavage sites. (B) Coomassie stained PAGE gel showing mHDx-1-TRX in lane 1, which appears as a single band. Cleavage by calpain 1 in lane 2 yields 3 smaller bands which correspond to the predicted products after cleavage at AA 8 and AA 15. Cleavage by EKMax in lane 3 yields 3 bands which correspond to the known cleavage sites. The N-terminal fragments generated by EKMax cleavage, which include the entire TRX tag and linker, are smaller than those generated by calpain cleavage indicating that calpain cleavage must occur within HDx-1. Lanes 4 and 5 are calpain 1 alone and EKMax alone respectively.

To determine if calpain directly or indirectly promotes clearance of mHDx-1 we incubated purified, recombinant calpain 1 and mHDx-1 protein *in vitro*. A thioredoxin tag (TRX) was fused to mHDx-1 to promote solubility. Cleavage at the predicted calpain recognition sites would result in N-terminal fragments consisting of the TRX tag and linker and N1-8 or N1-15 (Fig. 5a) As a control for cleavage within the TRX tag, mHDx-1-TRX was also incubated with EKMax, which removes the entire tag and linker sequence. Reactions containing either no protease or no HDx-1-TRX were used as controls. Reactions were separated by PAGE and visualized with coomassie to evaluate cleavage. In the absence of protease, mHDx-1-TRX protein appears as a single band of approximately 46 kDa. In the presence of calpain 1, mHDx-1-TRX is cleaved resulting in three smaller products (Fig. 5b). The smallest of these are 15.2 and 16.0 kDa which are very close to the predicted sizes for N1-8-TRX and N1-15-TRX of 15.1 and 15.9 kDa supporting cleavage at the predicted sites. These products are larger than those generated by EKMax cleavage indicating that calpain cleavage is occurring within mHDx-1. Immunoblotting with an antibody recognizing the N-terminus of Htt confirms that the 16.0 kDa cleavage fragment contains this domain supporting the predicted cleavage site (Fig. S2).

### V_L_12.3 binds to the putative calpain cleavage site at AA 15

**Figure 6:**
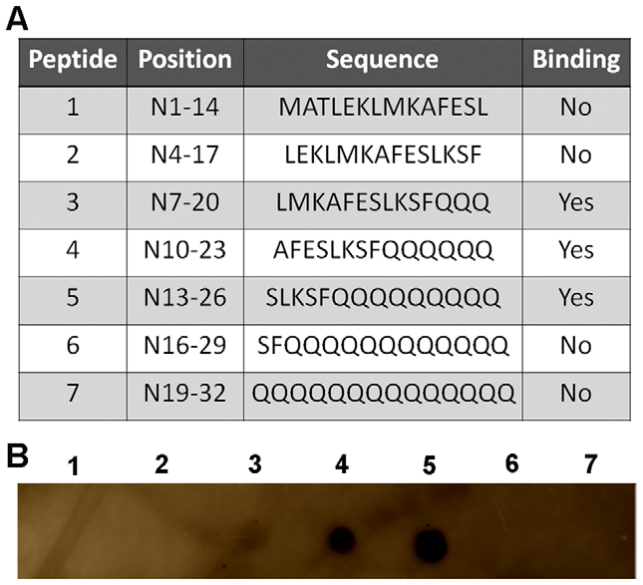
V_L_12.3 recognizes AAs 13-20 of HDx-1. Three AA stepped 14-mer peptides were spotted onto nitrocellulose and binding of V_L_12.3 was assessed. (A) Peptide table. (B) Dot blot showing binding of peptides 3, 4 and 5 illustrating that V_L_12.3 requires AAs 15-18 at the minimum and 13-20 at the maximum for recognition of Htt.

The iAb V_L_12.3 was selected for binding to an N1-20 AA fragment of HDx-1 [24], a domain that encompasses but is not limited to the predicted calpain cleavage sites. To determine the exact location of V_L_12.3 binding we used a 3 AA stepped peptide array binding assay (Fig. 6A). The results show that V_L_12.3 binds to peptides 3, 4 and 5 which are N7-20, N10-23 and N13-26, respectively (Fig. 6B). This demonstrates that V_L_12.3 requires AAs 15-18 at the minimum and 13–20 at the maximum for binding. Thus, V_L_12.3 binding would be expected to interfere with cleavage at AA 15 and possibly sterically hinder cleavage at AA 8.

### Blocking cleavage at AA 15 by V_L_12.3 binding prevents clearance of soluble mHDx-1

**Figure 7:**
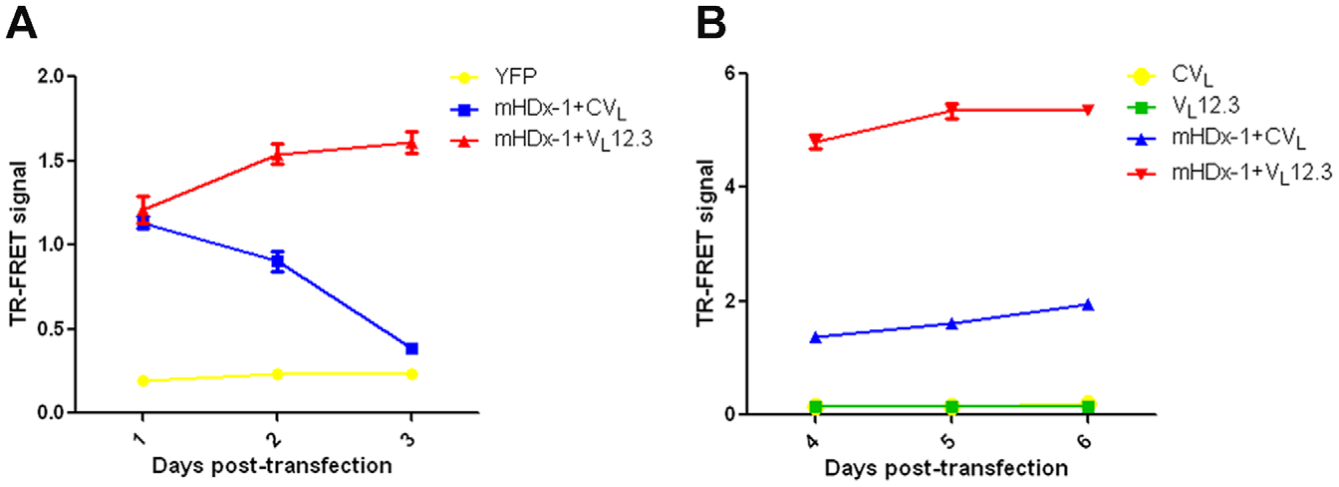
V_L_12.3 binding prevents turnover of HDx-1. (A) Organotypic brain slice cultures were co-transfected with mHDx-1 and V_L_12.3 or CV_L_, a control iAb. Soluble mHDx-1 protein level was assessed in lysates collected 1, 2 or 3 days post-transfection by TR-FRET. The level of mHDx-1 protein declines over time in the presence of CV_L_, but not in the presence of V_L_12.3 indicating impaired clearance. (B) Primary striatal and cortical neurons co-cultured with astroglia were transfected with iAb or mHDx-1 plus iAb. Soluble mHDx-1 protein level was assessed in lysates collected 4, 5 or 6 days post-transfection by TR-FRET. At these later time points, there is dramatically more mHDx-1 protein in the presence of V_L_12.3 as compared to CV_L_.

To determine the effect of compromising cleavage at AA 15 on mHDx-1 clearance, we performed a TR-FRET assay to measure soluble mHDx-1 levels in lysates of organotypic brain slice cultures biolistically transfected with mHDx-1 alone or with either V_L_12.3 or CV_L_, a control iAb. Soluble mHtt levels were compared 1, 2 and 3 days post-transfection by measuring the TR-FRET signal between a donor fluorophore-labeled antibody, 2B7, recognizing N1-17 of HDx-1, and an acceptor fluorophore-labeled antibody, MW1, recognizing polyQ [22]. This system is more suited to the measurement of reduced mHDx-1 turnover than the SNAP-tag fusion system described above, in which we observed no effect of V_L_12.3 on turnover rate. The brain slice culture system allows for longer experimental time frames during which, unlike the SNAP-tag system, significant normal mHDx-1 clearance is observed. This system also utilizes non-tagged mHDx-1 decreasing the likelihood that observed changes in mHDx-1 level are due to conformation or stability perturbations resulting from tag fusion. As expected, the level of soluble mHDx-1 in the presence of CV_L_ declines over time, reflecting normal clearance. In the presence of V_L_12.3, there is no change in mHDx-1 levels over time indicating a complete block of clearance (Fig. 7a). To extend our observation period even further, we have utilized a primary neuronal co-culture system consisting of striatal and cortical neurons as well as glia [21]. Primary neurons were transfected with iAb or mHDx-1 plus iAb and plated on a previously generated glial bed. Lysates were collected 4, 5 or 6 days later, and mHDx-1 protein level was assessed by TR-FRET. At these later time points, there is dramatically more mHDx-1 protein in the presence of V_L_12.3 than in the presence of CV_L_ (Fig. 7b). This suggests that clearance of mHDx-1 requires calpain cleavage at AA15, and that this cleavage event likely occurs upstream of CMA degradation.

## Discussion

Huntington's disease is a devastating neurodegenerative disease for which there is currently no disease modifying therapy. One of the difficulties with HD therapy development is the complex web of dysfunction resulting from the great many processes and pathways affected by mHtt protein in susceptible neurons. As a result, lowering the level of mHtt protein either by silencing expression or increased clearance remains a prime therapeutic approach for HD. For this reason, understanding the mechanism of mHtt degradation is important.

Huntingtin degradation involves numerous pathways, with differential toxicity regulated by post-translational modifications. Transgenic mice expressing mHtt that is resistant to caspase-6 cleavage at AA 586 do not develop the HD-like symptoms seen in their caspase-6-sensitive counterparts, despite the presence of other caspase cleavage products in the brain [9]. Phosphorylation of serine 421 shifts processing toward these less toxic products by inhibiting cleavage at AA 586 [11]. Moreover, calpain-resistant mHtt lacking the AA 469 and AA 536 cleavage sites is less toxic and aggregation-prone than calpain cleavage-sensitive mHtt [10]. Phosphorylation of serine 536 inhibits cleavage at AA 536, which also results in reduced toxicity [25]. Modifications of mHtt can also regulate non-protease degradation events. Phosphorylation of serines 13 and 16 increases proteasomal and lysosomal degradation of Htt in turn reducing toxicity. In *Drosophila*, presumably due to the absence of mammalian degradative machinery and mechanisms, this modification leads to increased toxicity due to accumulation of the more toxic phosphorylated form of mHtt [12]. A better understanding of the complex process of mHtt proteolysis could eventually lead to the development of therapeutics that shift processing toward less toxic pathways and/or enhance removal. Although the generation of N-terminal mHtt fragments by caspase and calpain cleavage has been previously characterized, the subsequent degradation of the highly toxic HDx-1 fragment has remained unclear.

With their high target specificity, iAbs are an ideal molecular tool for elucidating protein interactions and functions. For example, the 17 N-terminal AAs of HDx-1 are required for aggregate seeding and cytoplasmic retention [26], [27]. Blockade of this region by the binding of the iAb V_L_12.3 results in nuclear translocation and a potent inhibition of aggregation of HDx-1, illustrating the informative relationship between iAb effects and epitope function [14], [24]. Happ1 recognizes the proline rich region of HDx-1 and increases clearance of mutant but not wild type HDx-1 [14]. We have exploited this effect of Happ1 binding to gain insight into the mechanism of HDx-1 proteolysis.

Htt undergoes a variety of proteolytic processing steps including protease cleavage, proteasomal degradation, and lysosomal/autophagic degradation. In order to determine the initiating or rate-limiting step in mHDx-1 degradation, we tested inhibitors of each of these pathways in the presence and absence of Happ1 and evaluated mHDx-1 levels and turnover using the SNAP-tag method. One caveat of this method is that it requires the use of tagged HDx-1, which could alter HDx-1 conformation or stability. However, we obtained supportive evidence for the effect of V_L_12.3 on unlabeled HDx-1 using the entirely independent TR-FRET technique. Moreover, the stimulatory effect of Happ1 on mHDx-1 turnover labeled with the SNAP-tag is quite consistent with the significant lowering of mHDx-1-GFP levels by Happ1. An important control in our experiments is the use of mHDx-1ΔPRR, which lacks the Happ1 binding site. Levels of this protein are not affected by Happ1, indicating that the reduced mHDx-1 levels in the presence of Happ1 do not result from non-specific iAb actions such as activation of the un-folded protein response [28].

The proteasome inhibitors epoxomicin and lactacystin do not disrupt Happ1 stimulation of HDx-1 turnover, and Happ1 does not increase ubiquitination of HDx-1. These results indicate that Happ1 does not accelerate proteasomal degradation of Htt. We also find that 3-MA, an inhibitor of autophagosome formation and the macroautophagy pathway, does not interfere with Happ1-accelerated mHDx-1 degradation. In contrast, bafilomycin A1, a vacuolar-type H(+)-ATPase inhibitor that hinders lysosome-autophagosome fusion as well as disrupting lysosomal pH, prevents Happ1-induced changes in mHDx-1 clearance rate. Due to the lack of effect of 3-MA, it is unlikely that the action of bafilomycin A1 on autophagosome/lysosome fusion is responsible for disrupting Happ1 function. It is more likely that bafilomycin A1 disrupts Happ1 function by disrupting lysosomal pH. This indicates a role for CMA, which is an autophagosome-independent lysosomal degradation process, in Happ1-enhanced mHDx-1 clearance.

We next evaluated the sensitivity of the Happ1 effects to the caspase and calpain proteases. While caspase inhibition has no effect on Happ1 function, calpain inhibition is effective in blocking the ability of Happ1 to both decrease the level of soluble mHDx-1 and increase its turnover. These results indicate that Happ1 likely increases mHDx-1 clearance through enhanced calpain cleavage, which is particularly interesting because of the lack of a known calpain cleavage site in HDx-1. Calpain inhibitor I has, however, been reported to cause accumulation and increased aggregation of N-terminal Htt fragments including HDx-1, and calpain 1 is known to increase degradation of these fragments in the lysates of transfected PC12 cells [29]. Taken together, these results indicate that calpains participate either directly or indirectly in the degradation of mHDx-1.

Analysis of the AA sequence of human HDx-1 using the web application SitePrediction identifies AAs 12-17 as having the highest degree of specificity for both calpain 1 and calpain 2, with a secondary recognition site at AA 5-10. Cleavage at these sites, which is not predicted to be modulated by increasing polyQ length, would result in the removal of 15 of the 17 N-terminal AAs of Htt, effectively removing the N-terminus. The N-terminus of Htt is the site of many post-translational modifications including phosphorylation, acetylation and sumoylation [30], [31]. In the wt protein, this domain adopts an amphipathic alpha-helical structure, which associates with cellular membranes [26], [32]. The predicted cleavage site, AA15, is part of the charged face of this helix and would therefore be exposed, but the tightly packed nature of this secondary structure could inhibit cleavage. In the context of expanded polyQ, the secondary structure of the N-terminus is significantly disrupted [33] leading to an un-coiling, which could increase exposure of the predicted cleavage site. This could explain the differential action of Happ1 on mutant and wtHtt. It is interesting to note that phosphorylation of this putative calpain cleavage site is known to increase mHDx-1 nuclear localization followed by degradation [12] and that removal of the N-terminus is known to increase nuclear localization [26], [27].

Calpain I cleaves purified mHDx-1 *in vitro*, generating cleavage products of the expected sizes, showing that this protease can act directly on HDx-1 and supporting the site prediction. We employed iAb blockade of the N-terminal 12-17 AA site to determine the importance of cleavage here in the degradative process. V_L_12.3 was raised against a peptide of AAs 1–20 of Htt and therefore binds somewhere in this region [24], which includes but is not limited to the putative calpain cleavage sites identified here. Peptide array epitope mapping shows that V_L_12.3 binding requires at a minimum, AAs 15–18 of HDx-1 for binding, a region that includes the putative calpain cleavage site at AA 15. As V_L_12.3 binding is known to prevent interactions of HDx-1 that require this domain, such as aggregate seeding and cytoplasmic retention [14], it is reasonable to postulate that V_L_12.3 would also compromise cleavage here. If calpain cleavage at this site is involved in the degradation of mHDx-1, V_L_12.3 binding would be expected to reduce turnover. We have previously shown that V_L_12.3 binding has no effect on mHDx-1 protein level or turnover rate in cultured 293 and ST14A cells, respectively [14]. These systems are, however, temporally constrained by the toxicity of transfection reagents and mHDx-1 as well as cell proliferation. These factors limit our experimental time frame to 24 Hrs, an interval in which we observe very little normal mHDx-1 clearance, and any decreases in clearance may be below the sensitivity threshold of the assays. As a result, these systems, although sufficient for evaluating increased turnover, are inadequate for evaluating decreased turnover. Moreover, these systems lack differentiated neurons and connectivity, which are integral to HD pathology and require the use of GFP or SNAP-tag fusions, which could alter mHDx-1 stability. In an effort to overcome these caveats, we used a TR-FRET assay to evaluate the effect of V_L_12.3 on non-tagged mHDx-1 clearance in biolistically co-transfected brain slice explants and in primary corticostriatal neuronal co-cultures, which allow longer experimental time frames of up to 3 and 6 days, respectively, in more relevant, partially intact neuronal systems. In these systems in the presence of CV_L_, an iAb that does not bind HDx-1, the level of mHDx-1 protein appears to decline over the first three days, reaching a plateau that is maintained for the subsequent 3 days, reflecting normal turnover. Conversely, during the observed time period there is no change in mHDx-1 level in the presence of V_L_12.3, demonstrating a lack of normal turnover when the putative calpain cleavage site is bound by the iAb. These results suggest that calpain-mediated removal of the N-terminus of mHDx-1, which is likely followed by CMA degradation, is required for clearance of this toxic protein and that selective regulation of this cleavage event could prove beneficial in the treatment or prevention of HD.

## Supporting Information

Figure S1 **There are predicted calpain cleavage sites at AAs 12-17 and 5-10 of HDx-1 with high specificity for calpains 1 and 2.** Human HDx-1 sequence was analyzed using the web tool SitePrediction for predicted calpain 1 and 2 cleavage sites. This analysis determined that AAs 12-17 is predicted to have the greatest specificity for both proteases. There is a secondary predicted cleavage site at AA 5-10 that is also predicted to be highly specific for both calpain 1 and 2.
(TIF)

Figure S2 **The 16.0 kDa calpain cleavage fragment contains the Htt N-terminus.** HDx-1 Q46 fused to thioredoxin (mHDx-1-TRX) was incubated alone or with purified calpain 1 *in vitro*, separated by PAGE and transferred to nitrocellulose membrane. Immunoblotting with an antibody recognizing the N-terminus of Htt reveals that the 16.0 kDa cleavage product contains this domain.
(TIF)

## References

[1] The Huntington's Disease Collaborative Research Group (1993). A novel gene containing a trinucleotide repeat that is expanded and unstable on Huntington's disease chromosomes.. Cell.

[2] Imarisio S, Carmichael J, Korolchuk V, Chen C-W, Saiki S (2008). Huntington's disease: from pathology and genetics to potential therapies.. Biochem J.

[3] Gil JM, Rego AC (2008). Mechanisms of neurodegeneration in Huntington's disease.. Eur J Neurosci.

[4] Qin Z-H, Gu Z-L (2004). Huntingtin processing in pathogenesis of Huntington disease.. Acta Pharmacol Sin.

[5] Landles C, Sathasivam K, Weiss A, Woodman B, Moffitt H (2010). Proteolysis of Mutant Huntingtin Produces an Exon 1 Fragment That Accumulates as an Aggregated Protein in Neuronal Nuclei in Huntington Disease.. J Biol Chem.

[6] Ratovitski T, Gucek M, Jiang H, Chighladze E, Waldron E (2009). Mutant Huntingtin N-terminal Fragments of Specific Size Mediate Aggregation and Toxicity in Neuronal Cells.. J Biol Chem.

[7] Majumder P, Raychaudhuri S, Chattopadhyay B, Bhattacharyya NP (2007). Increased caspase-2, calpain activations and decreased mitochondrial complex II activity in cells expressing exogenous huntingtin exon 1 containing CAG repeat in the pathogenic range.. Cell Mol Neurobiol.

[8] Todde V, Veenhuis M, van der Klei IJ (2009). Autophagy: Principles and significance in health and disease.. BBA - Mol Basis Dis.

[9] Graham RK, Deng Y, Slow EJ, Haigh B, Bissada N (2006). Cleavage at the caspase-6 site Is required for neuronal dysfunction and degeneration due to mutant huntingtin.. Cell.

[10] Gafni J, Hermel E, Young JE, Wellington CL, Hayden MR (2004). Inhibition of calpain cleavage of huntingtin reduces toxicity: accumulation of calpain/caspase fragments in the nucleus.. J Biol Chem.

[11] Warby SC, Doty CN, Graham RK, Shively J, Singaraja RR (2009). Phosphorylation of huntingtin reduces the accumulation of its nuclear fragments.. Mol Cell Neurosci.

[12] Thompson LM, Aiken CT, Kaltenbach LS, Agrawal N, Illes K (2009). IKK phosphorylates huntingtin and targets it for degradation by the proteasome and lysosome.. J Cell Biol.

[13] Southwell AL, Patterson PH (2010). Antibody therapy in Neurodegenerative disease..

[14] Southwell AL, Khoshnan A, Dunn DE, Bugg CW, Lo DC (2008). Intrabodies binding the proline-rich domains of mutant huntingtin increase its turnover and reduce neurotoxicity.. J Neurosci.

[15] Southwell AL, Ko J, Patterson PH (2009). Intrabody Gene Therapy Ameliorates Motor, Cognitive, and Neuropathological Symptoms in Multiple Mouse Models of Huntington's Disease.. J Neurosci.

[16] Verspurten J, Gevaert K, Declercq W, Vandenabeele P (2009). SitePredicting the cleavage of proteinase substrates.. Trends Biochem Sci.

[17] Kent WJ, Sugnet CW, Furey TS, Roskin KM, Pringle TH (2002). The human genome browser at UCSC.. Genome Res.

[18] Bennett MJ, Huey-Tubman KE, Herr AB, West AP, Ross SA (2002). A linear lattice model for polyglutamine in CAG-expansion diseases.. PNAS.

[19] Mende-Mueller LM, Toneff T, Hwang S-R, Chesselet M-F, Hook VYH (2001). Tissue-Specific Proteolysis of Huntingtin (htt) in Human Brain: Evidence of Enhanced Levels of N- and C-Terminal htt Fragments in Huntington's Disease Striatum.. J Neurosci.

[20] Lo DC, McAllister AK, Katz LC (1994). Neuronal transfection in brain slices using particle-mediated gene transfer.. Neuron.

[21] Kaltenbach LS, Bolton MM, Shah B, Kanju PM, Lewis GM (2010). Composite primary neuronal high-content screening assay for Huntington's disease incorporating non-cell-autonomous interactions.. J Biomol Screen.

[22] Weiss A, Abramowski D, Bibel M, Bodner R, Chopra V (2009). Single-step detection of mutant huntingtin in animal and human tissues: A bioassay for Huntington's disease.. Anal Biochem.

[23] Jansen LET, Black BE, Foltz DR, Cleveland DW (2007). Propagation of centromeric chromatin requires exit from mitosis.. J Cell Biol.

[24] Colby DW, Garg P, Holden T, Chao G, Webster JM (2004). Development of a human light chain variable domain (VL) intracellular antibody specific for the amino terminus of huntingtin via yeast surface display.. J Mol Biol.

[25] Schilling B, Gafni J, Torcassi C, Cong X, Row RH (2006). Huntingtin phosphorylation sites mapped by mass spectrometry: modulation of cleavage and toxicity.. J Biol Chem.

[26] Atwal RS, Xia J, Pinchev D, Taylor J, Epand RM (2007). Huntingtin has a membrane association signal that can modulate huntingtin aggregation, nuclear entry and toxicity.. Hum Mol Genet.

[27] Rockabrand E, Slepko N, Pantalone A, Nukala VN, Kazantsev A (2007). The first 17 amino acids of Huntingtin modulate its sub-cellular localization, aggregation and effects on calcium homeostasis.. Hum Mol Genet.

[28] Schroder M, Kaufman RJ (2005). The mammalian unfolded protein response.. Ann Rev Biochem.

[29] Ratovitski T, Nakamura M, D'Ambola J, Chighladze E, Liang Y (2007). N-terminal proteolysis of full-length mutant huntingtin in an inducible PC12 cell model of Huntington's disease.. Cell Cycle.

[30] Steffan JS, Agrawal N, Pallos J, Rockabrand E, Trotman LC (2004). SUMO modification of duntingtin and Huntington's disease pathology.. Science.

[31] Aiken CT, Steffan JS, Guerrero CM, Khashwji H, Lukacsovich T (2009). Phosphorylation of Threonine 3: Implications for huntingtin aggregation and neurotoxicity.. J Biol Chem.

[32] Kim MW, Chelliah Y, Kim SW, Otwinowski Z, Bezprozvanny I (2009). Secondary Structure of Huntingtin Amino-Terminal Region.. Structure.

[33] Thakur AK, Jayaraman M, Mishra R, Thakur M, Chellgren VM (2009). Polyglutamine disruption of the huntingtin exon 1 N terminus triggers a complex aggregation mechanism..

