# Supplementary figures and images for: Perturbation with Intrabodies Reveals That Calpain Cleavage Is Required for Degradation of Huntingtin Exon 1
Source: PLoS One. 2011 Jan 31;6(1):e16676. doi: 10.1371/journal.pone.0016676 (PMC3031625; doi:10.1371/journal.pone.0016676)

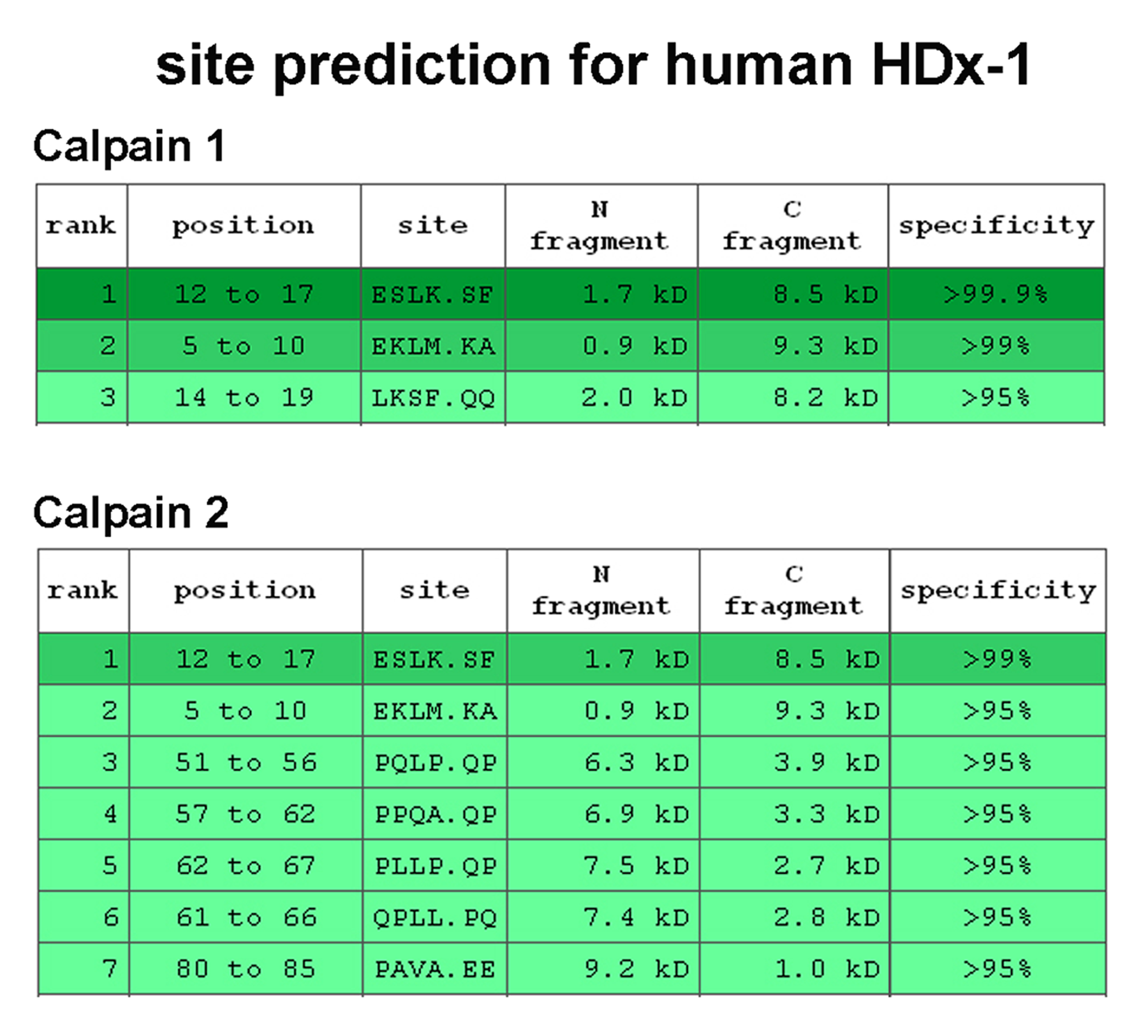

Supplement: Figure S1 — There are predicted calpain cleavage sites at AAs 12-17 and 5-10 of HDx-1 with high specificity for calpains 1 and 2. Human HDx-1 sequence was analyzed using the web tool SitePrediction for predicted calpain 1 and 2 cleavage sites. This analysis determined that AAs 12-17 is predicted to have the greatest specificity for both proteases. There is a secondary predicted cleavage site at AA 5-10 that is also predicted to be highly specific for both calpain 1 and 2. (TIF) [file pone.0016676.s001.tif]

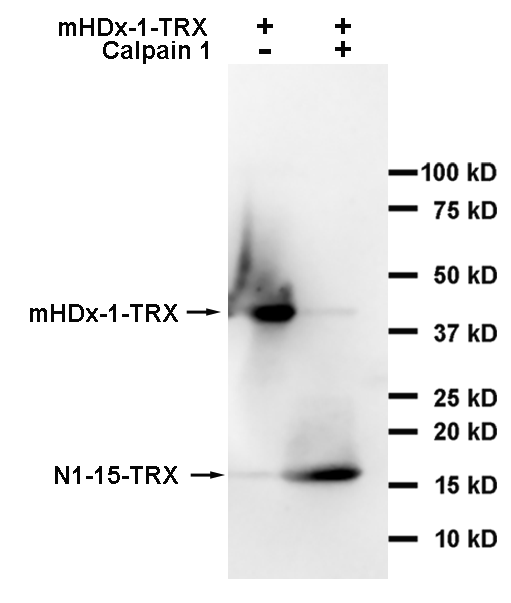

Supplement: Figure S2 — The 16.0 kDa calpain cleavage fragment contains the Htt N-terminus. HDx-1 Q46 fused to thioredoxin (mHDx-1-TRX) was incubated alone or with purified calpain 1 in vitro, separated by PAGE and transferred to nitrocellulose membrane. Immunoblotting with an antibody recognizing the N-terminus of Htt reveals that the 16.0 kDa cleavage product contains this domain. (TIF) [file pone.0016676.s002.tif]
